# Mother of One to Mother of Two: A Textual Analysis of Second-Time Mothers’ Posts on the BabyCenter LLC Website

**DOI:** 10.3389/fpsyg.2022.859085

**Published:** 2022-04-25

**Authors:** Emma Beyers-Carlson, Sarita Schoenebeck, Brenda L. Volling

**Affiliations:** ^1^Department of Psychology, University of Michigan, Ann Arbor, MI, United States; ^2^School of Information, University of Michigan, Ann Arbor, MI, United States

**Keywords:** second-time mothers, BabyCenter, birth of a sibling, firstborn children, pregnancy, social media, online parenting

## Abstract

Mothers use online resources frequently to obtain information on pregnancy, birth, and parenting. Yet, second-time mothers may have different concerns than first-time mothers given they have a newborn infant and another child at home. The current study conducted an on-line textual analysis of the posts of second-time mothers during pregnancy and the first months postpartum on the BabyCenter LLC website, one of the largest online parenting communities. Latent Dirichlet Allocation (LDA) analysis on roughly 16,000 posts to BabyCenter birth clubs in 2017 by approximately 4,000 users revealed second-time mothers relied on the online support of the BabyCenter community to share and discuss topics of pregnancy, birth, and child rearing. Second-time mothers also raised questions about preparing their firstborn children for a new baby sibling, how they would care for two children, whether they would love the second one as much as the first, and how the second child would change family dynamics. Future research needs to recognize that second-time mothers may have distinct concerns surrounding the birth of their second baby, and antenatal education and parenting classes may need to be modified to be more inclusive of these women’s needs and perspectives. Online parenting communities offer avenues to support women as they make the transition from one child to two and may provide targeted opportunities to disseminate evidence-based practices that can assist these women and their children.

## Introduction

The rise of the internet introduced a drastic change in the ways that parents used information and interacted with others (Shirky, 2008). Though technology use is increasing, in general (Martin and Robinson, 2007; National Telecommunications and Information Administration, 2011), pregnant women and mothers have been particularly active Internet users. Thus, many women have turned to the online world for pregnancy and parenting advice, to find social support from other mothers, and to acquire information on infant health and development (Sarkadi and Bremberg, 2004; Daneback and Plantin, 2008; Plantin and Daneback, 2009; Dworkin et al., 2013). Further, online communities provide an environment of anonymity and disinhibition, which can create a safe space for women to navigate the online world as a social outlet to discuss their pregnancies and difficult parenting issues or to share concerns about their role as mothers without revealing their true identities (Schoenebeck, 2013).

The transition to the second child is one such area that many mothers may discuss online. Nearly 80% of families in the U.S. have at least two children, making the birth of a second child a frequent occurrence for many women. Though the transition to siblinghood (i.e., when only children become older siblings) has been studied in offline contexts (e.g., Dunn et al., 1981; Dunn and Kendrick, 1982; Stewart, 1990; Volling et al., 2017), there are no investigations into how mothers discuss the transition from one to two children in an online setting. Because there are few resources available to women making the transition from one child to two (Beyers-Carlson and Volling, 2017), soon to be second-time mothers may turn to the internet for information on how best to prepare their firstborn children and themselves as the birth of the baby sibling approaches. Child-rearing books and online information often portray the transition as a time of disruption filled with sibling rivalry and problematic child behaviors (Boyd, 2009; Edwards, 2010; Leach, 2010; Dais, 2016), even though recent research indicates that most children evince little disruptive behavior after the birth of a baby sibling. Even if there is an increase in problem behaviors, it is often short-lived (Volling et al., 2017). This does not mean, however, that the transition is less challenging for mothers. In her book, *Three shoes, one sock and no hairbrush: Everything you need to know about having your second child*, Abrams (2001) acknowledged the myth of the mother with only one child by stating “Having cared for one baby already, we may not need to be told how to look after a second *baby*, but what many women badly need is information about looking after *two children*” (p. 16). As such, there is a need to learn more about what concerns second-time mothers discuss online so that others can provide support and assistance to mothers going through the transition.

There has been little interest in the perinatal concerns of second-time mothers, with most extant studies based primarily on qualitative interviews with a small number of women to get a sense of what mothers discuss when expecting their second child. Many of these early studies, however, were published well over 30 years ago. Further, many of the interviews were with women seeking assistance with parenting difficulties or psychological counseling so the concerns raised by these women may be more serious and challenging than what may be found in a larger, more representative sample of second-time mothers. As such, information from these interviews can result in various self-report biases (Van de Mortel, 2008). The current research relied on naturalistic discussions shared by second-time mothers in an online context where they can be pseudonymous, which may reduce some self-presentational biases (Ammari et al., 2018). The main goal of the present investigation was to explore the topics second-time mothers discussed online both before and shortly after the birth of their second baby by focusing on on-line posts on the BabyCenter LLC website, one of the largest parent-focused sources of on-line information about pregnancy, birth, and parenting.

Although first-time mothers or mothers of twins may also have similar or different concerns than those raised by second-time mothers, we focus this review on second-time mothers given that they are often overlooked in the literature on pregnancy and the postpartum. First-time motherhood and even mothers of multiples are often recognized as having unique needs that need to be addressed by professionals (e.g., Feldman and Eidelman, 2004; Law et al., 2019; Crugnola et al., 2020). The current report allows the voices of second-time mothers to be heard so that both researchers and practitioners include these women when designing services and interventions for mothers. The prior qualitative research indicated three primary areas of concern that appeared relevant to second-time mothers and we will review each briefly here: (1) anxieties about pregnancy and relations with the first child; (2) being a mother to two children, a baby and an older child; and (3) changes in family dynamics, social support and work-family balance.

Mothers often reported feelings of stress around their changing roles as a mother of one to a mother of two and conflicted feelings regarding changes in their relationship with the first child (Pridham et al., 1982; Richardson, 1983a). They often expressed feelings of guilt, worried that they were betraying their first child by having a second child (Jenkins, 1976; Moss, 1981; Krieg, 2007), and reported grieving over the loss of their exclusive relationship with their firstborn (Rubin, 1976; Richardson, 1983b; Walz and Rich, 1983; Young et al., 1983; Fisher, 1987). Many mothers also questioned their ability to emotionally care for two children and specifically, anxiety around their capacity to love two children equally (Jenkins, 1976; Mercer, 1979; Walz and Rich, 1983).

Because second-time mothers were pregnant, they also talked frequently about the physical aspects of pregnancy, labor, and delivery, such as physical discomfort, fatigue, and the pain that accompanies delivery and birth (Westbrook, 1978; Norr et al., 1980; Hiser, 1987). They often acknowledged that no two pregnancies were ever the same and would often compare their second pregnancy with what they experienced during their first. Even though mothers had given birth to a child once before and were familiar with labor and delivery, many still raised concerns about the course of their current pregnancy, the possibility of delivery complications, and in the postpartum period, the health of the newborn infant.

Second-time mothers also spoke specifically about the adjustment of their first children to the arrival of a second baby, and as a result, wanted more information on how best to prepare their children for the infant’s birth. Some mothers feared that their older children would not accept their new siblings or might even harm the infant (Richardson, 1981, 1983b; Fisher, 1987). Many mothers wanted to know how to prevent sibling rivalry and jealousy of the new baby, wondering if the spacing between the children was too far apart or too close together, and how this would affect the developing sibling relationship (Mercer, 1979; Krieg, 2007). They also spoke of their motivations for having a second child, such as hoping the new baby would be a companion for the first (Crawford and Boyer, 1984). These mothers often actively sought out any information available about parenting two children and how to create new caregiving and family routines with two children (Hiser, 1987; Jordan, 1989; O’Reilly, 2004). Some mothers questioned their abilities as a mother to care for two young children—a new baby and a toddler—or whether they could meet the needs of both children without neglecting one over the other (Jenkins, 1976; Fisher, 1987). Finally, several mothers discussed their attempts to promote their children’s maturity (e.g., encouraging the older child to dress themselves; toilet training) before the infant was born to ease the transition (Walz and Rich, 1983; Fisher, 1987), hoping that more independence and autonomy on the part of the first child would help alleviate the demands of caring for two young children.

Mothers in these earlier studies also commented on changes in their relationships with their partners (Richardson, 1981; Ulrich, 1981; O’Reilly, 2004; Krieg, 2007), noting specifically the division of child-care and household labor now that there would be two children in the family with different needs and schedules. Mothers expressed a desire for more partner support for child-care and housework (Affonso et al., 1988; Jordan, 1989; Nichols et al., 2007). Several mothers noted issues with their support networks and how some extended family members expressed less enthusiasm over the second pregnancy than was the case with the first pregnancy, and how this was a source of dismay (Norr et al., 1980; Jordan, 1989; O’Reilly, 2004). Mothers also expressed a desire for material support (e.g., caregiving assistance, meal preparation), similar to what they received with the birth of their first child, but questioned if they would receive such support from family and friends as their social network appeared to believe there was less need for assistance the second time given mothers had already birthed and parented one child (Larsen, 1966; Westbrook, 1978). Finally, mothers discussed the logistics of managing work and family life with two young children, including planning new routines to encompass the schedules of both children, managing finances now that there were two children, handling the increased workload with the addition of a new baby, and whether they would have enough maternity leave available to spend time with the infant (Larsen, 1966; Walz and Rich, 1983; Nichols et al., 2007; Barnes, 2013; Frost and Rodriguez, 2015).

Because information on parenting and pregnancy is now readily available to parents via the internet, and several child-rearing books have been published on how to manage the transition with a second child (e.g., Leonard, 2000; Edwards, 2010; Cooper-Abbs, 2013; Dais, 2016), one question to ask is whether second-time mothers discuss similar issues about preparedness for the birth of their second child today as did mothers interviewed for these earlier studies nearly 30 years ago. Given the many social media outlets and technological changes accompanying the increase in internet use, parenting websites, and phone apps, mothers in the early twenty first century may be more satisfied and less worried about these issues and feel better prepared. Knowing whether second-time mothers continue to discuss these issues and express some of the same anxieties surrounding the pregnancy, birth, and early parenting of a second baby, will provide much needed information for practitioners wishing to assist women undergoing the transition from one child to two. The current study was a preliminary investigation exploring the online world of second-time mothers using birth clubs on the BabyCenter LLC website.

## Materials and Methods

### BabyCenter LLC

Internet sites that include anonymous forums for parents provide data-rich media for acquiring information about issues surrounding pregnancy and parenting because they aggregate candid questions and conversations between thousands of users. The anonymous nature of online message boards provides an environment for mothers to discuss issues more freely and openly, and where they may be willing to broach topics and reveal anxieties that they may not have felt comfortable sharing with family members or even health care professionals for fear they would be judged (Schoenebeck, 2013). BabyCenter LLC is an online parenting forum that allows a unique opportunity to explore women’s experiences during the transition to the second child by taking advantage of a social media context to understand the experiences of thousands of users.

BabyCenter LLC is a website targeting U.S. parents through pregnancy, birth and early development.^1^ Initially founded in 1997, the site was later acquired by the Johnson and Johnson family of companies in 2001 and sold again in 2019 to the Everyday Health Group, a digital media company that produces health-related content. The BabyCenter website^2^ describes the site as “the world’s number one digital parenting resource, with content that reaches more than 100 million people monthly. In the United States, 7 in 10 new and expectant moms online use BabyCenter.” The website is divided into five major areas: (1) *Expert Advice*, which includes topics, tools, and resources around pregnancy and parenting during the childhood years that are written by BabyCenter staff and reviewed by the BabyCenter Advisory Board (a team of doctors and professionals in a variety of medical, physical, and emotional health fields); (2) a *Blog*, which comprises articles around parenting written by freelance writers for BabyCenter; (3) *Products and Gear*, a section which includes BabyCenter vetted and “Mom Picks” (the top items chosen by users on the site) paraphernalia for childrearing; (4) *Mission Motherhood*, a section devoted to BabyCenter’s non-profit work; and (5) *Community*, a pseudonymous message board for parents, which contributed the primary data for this paper.

BabyCenter’s Community forum is primarily text-based and is organized into (a) *Birth Clubs*, message boards devoted specifically to people expecting a child in a specific month and year (e.g., January 2017 Birth Club); (b) *Groups*, discussion boards centered around specific topics (e.g., family life, breastfeeding support, ultrasounds); (c) *Mom Answers*, in which a BabyCenter user can post a question and have other mothers in the community answer; and (d) *Photo Clubs*, forums in which users only post pictures centered around specific topics. Any visitor to the site can view public posts in birth clubs and groups, but users who want to post content to or belong to private groups must create an account with a username and a password. BabyCenter is a pseudonymous site, which means that many users choose pseudonyms for their usernames (rather than their actual names). Text data for the current report were culled from community discussions from two second-time parent birth clubs spanning 2016 and 2017.

BabyCenter’s birth clubs specific to second-time parenting were our primary focus and allowed a unique opportunity to obtain online text data to explore the topics and sentiments mothers discussed around the transition to the second child. The current study assessed the themes of second-time motherhood using Latent Dirichlet Allocation (LDA) to extract lexical groups (LG) using the text from thousands of posts that reflect different topics. LDA modeling has been successfully applied to investigate subjects of interest relevant to psychology and children’s development from a variety of social media settings. For example, Xu et al. (2012) used LDA models to study bullying through social media, and Chancellor et al. (2016) used LDA to obtain information on mental illness severity from online communities. Ammari et al. (2018) used LDA to investigate the topics parents discussed on Reddit, another online community, by extracting LG’s from parent posts. The primary aim of this investigation was to isolate predominant topics expressed by second-time mothers on BabyCenter LLC over several months of their pregnancies (pre-birth) and into the early months postpartum to get an initial sense of what these women discussed in their online communities of support. We were also interested in seeing if these topics and themes were similar or dissimilar to topics discussed by second-time mothers nearly three decades earlier in the small qualitative interview studies.

Similar to other online parent message boards, content on BabyCenter LLC is archived so it is possible to crawl (i.e., use automated computer programs) content within specific periods of time. Though the data were publicly available, there are still ethical considerations when using on-line text data, including how the use of the data might benefit or harm the people who shared it (Feisler and Proferes, 2018). In this article, we used recommended guidelines for working with publicly available on-line media as recommended by the APA Board of Scientific Affairs Advisory Group on Conducting Research on the Internet (see also Kraut et al., 2004). This included anonymizing the specific birth club months from which we collected data by noting Month A and Month B, instead of the exact months and years, and provided approximations of the actual number of posts and users in each birth club rather than exact figures. We also lightly anonymized quotations drawn from the boards as exemplars to reduce the likelihood of revealing the identity of the poster, even though most, if not all, posters used pseudonyms rather than revealing their true identities; one of the reasons posts on these sites provide a rich opportunity to uncover what women discuss in a pseudonymous forum. The risks of using these data were no more than the risks women were taking in posting such information publicly on these forums. Finally, one of the benefits of this research was to acquire information to support second-time mothers and allow practitioners insights into the thoughts and feelings of this often overlooked group of women.

Parents join these birth clubs based on the birth month that the baby is due. For example, a parent who has an older child and was due with the second child in November 2015 could join a birth club for “November 2015 Second-Time and Beyond Parents.” We collected text data for the current report from two sequential birth clubs, labeled “Month A” (roughly 7,000 posts, 3,000 threads, and 2,000 unique users at the time of data collection) and “Month B” (roughly 8,000 posts, 3,000 threads, and 2,000 unique posting users) at the time of data collection in 2017, which were combined to create a second-time parenting dataset (roughly 16,000 comments, 5,000 threads, and 4,000 unique posting users). To isolate LG’s specific to the prenatal period, including when the baby sibling was born, and the postnatal period following the birth, the text data set was divided into pre-birth (the months before the baby was due) and post-birth time frames (the due date month and subsequent 8 months after the birth), spanning from 2016 to 2017. The dataset included comments, usernames (pseudonyms), and the timestamp of the specific comment, but did not include any other identifying information (e.g., home address, gender). Some users appeared to occasionally use part of their given name in their username but this was rare. We used a set of common English stopwords included in the widely used nltk (Natural Language Toolkit) of the Python library. Stopwords (e.g., “the,” “is,” “are”) were removed from the textual dataset and we ignored words with little relevance for analytical purposes such as “anything.”

Due to the pseudonymous nature of the site, we cannot know the demographics of the BabyCenter Community with certainty but users in the Community appeared to be primarily based in the U.S. and had groups devoted specifically to certain states or areas of the country. BabyCenter LLC is marketed broadly as a parenting forum, including sections specifically “Just for Moms” or “Just for Dads,” but its user base at the time of this investigation, particularly for the Community section, appeared to be mainly populated by mothers, evidenced by the predominance of mother-centered topics and identities (e.g., usernames, pregnancy, breast-feeding). As such, we use mother-coded language to describe the parent responses throughout the remainder of this paper.

### Latent Dirichlet Allocation Topic Modeling

The LDA model (Blei et al., 2003) was used to extract latent topics (e.g., co-occurring sets of terms in a text corpus; Bansal, 2015) from text documents created from crawled data of the second-time parenting birth clubs. LDA is used with text data for topic mining and the analysis includes a three-level Bayesian model that uses machine learning to generatively and probabilistically identify topics present in a body of text to detect patterns present in the data. As such, it represents text documents created from the crawled data as a collection of topics that are exemplified as a body of words gleaned from the text corpora that probabilistically match that topic (Blei et al., 2003; Chen, n.d.). LDA uses a set of algorithms. As is often the case in papers that use machine learning to study social behavior, it is not useful to include the algorithms themselves but instead, to describe the intuition behind what they do. LDA is an approach to detecting patterns in large amounts of data using algorithms. LDA works by identifying patterns across words contained in a document, and by associating groups of words with topics. We chose LDA because it does not require labeled data, which social media data rarely are, and because it is a robust approach used across computational and social contexts. LDA does not utilize prior investigator-based expectations about the topics that may be present. Instead, LDA allows all parameters to be free, does not impose any prior expectations on part of the researcher, and generatively isolates topics present in the data that may have been previously unknown to the researcher (Zhai, n.d.).

We trained two independent LDA models (i.e., two separate bodies of text from the website) using the Python gensim package corresponding to two time periods: (a) pre-birth and (b) post-birth., which allowed us to isolate what second-time mothers discussed during pregnancy and the postpartum period. The output of each LDA model contained a set of topics, each represented by a group of tokenized keywords, which we refer to as lexical groups (LG)—see Supplementary Tables 1, 2 for a complete list of the LG’s generated for this study. Given the exploratory and preliminary nature of this research and the fact that few studies consider second-time mothers, the research team specified the models to output no more than 20 topics per the two lexical bodies of text (pre- and post-birth) for a total of 40 sets.

### Text Analysis and Identification of Content

We used an inductive approach to analyze the 20 pre- and 20 post-birth LG’s. Two members of the research team, including the first author and a trained research assistant, independently examined each LG and provided a topic label that they believed best described the tokenized words of each LG extracted from the LDA. Inter-rater reliability across the two coders using kappa was *k* = 0.79. Inter-rater reliability is a means of calculating agreement between independent coders and is a measure of the extent to which coders or raters assign the same score to the same variable, or in our case, the same descriptive label to an LG. In the current work, two coders reviewed the LG’s from the LDA independently and each came up with a descriptive label for each LG. These labels were then reviewed and percent agreement (# of agreements/# of agreements + disagreements) was calculated as a means of interrater agreement. Cohen’s kappa statistic is a measure of interrater reliability that takes chance agreement into consideration given that some coders may agree simply by chance. Thus, percent agreement can inflate the level of interrater reliability. Kappa coefficients can be interpreted similar to a correlation with a range from -1 to 1 although it is very rare that a measure of agreement would be less than 0. According to Landis and Koch (1977), kappa values can be interpreted as follows: 0–0.20 slight agreement; 0.21–0.40 fair; 0.41–0.60 moderate; 0.61–0.80 substantial; and 0.81–1.0 perfect. The Kappa value in our work is 0.79 which means that interrater reliability was substantial.

Next, the team, which included the third author, met as a group to discuss the resulting LG descriptors, refined the labels for each LG, and through consensus, decided on the final labels for each LG topic. Although the research team used a two-step approach to obtain reliable labels, we must acknowledge that the labels are based on a subjective appraisal of each LG. Throughout the results we provide quotes and examples of posts that best exemplify the LG topics, which can also be found in Supplementary Tables 1, 2.

## Results

### Timeline for Pre-birth Topics Posted by Second-Time Mothers

Given the manner in which the analyses were conducted and due to the unique longitudinal nature of the dataset spanning across time from the first prenatal months to post-birth, the 20 identified LGs isolated from the pre-birth data and the 20 LGs isolated from the post-birth data are based on a temporal timeline and are presented in the results temporally in the order that they were uncovered from pregnancy throughout the postpartum. The 20 LG topics posted during pregnancy and before the infant’s birth are summarized along a timeline from early pregnancy to late pregnancy, similar to other presentations of LG’s from parenting websites (e.g., Ammari et al., 2018). The full summary of LG’s for the pre-birth modeling along with an exemplar post can be found in Supplementary Table 1 of Supplementary materials. Early in the pregnancy, mothers relayed stories of announcing their pregnancy to family and friends and gave feedback to each other on the best time to do so. They also discussed concerns about the safety of certain substances during pregnancy (e.g., are certain vitamins safe for use during pregnancy?). Another topic reflected the fear that some mothers had that they would not love the new baby as much as their first child, and several expressed guilt both over the change in their relationship with the firstborn and over the mixed emotions surrounding the new pregnancy. Although mothers mentioned they were happy for the birth of their second baby, they also noted the feelings of loss over the exclusive relationship with their firstborn and worried if it was normal for them to feel anxious about whether they would be able to love their second child as much as their first. This was exemplified by the following post from one mother:

*Hello everyone, I’m a little conflicted about the way I feel about this pregnancy. Me and my husband have a 5 year old wonderful girl. I just finished school and we planned to get pregnant right after I graduated. That was in May. Being 26, I got pregnant the first try. I just found out on Sunday and I got very nervous. I thought I was going to react differently, and be over the moon happy. But when I found out, I felt happy but just a little sad.maybe. I mean, we had been planning this for a long time and my girl is excited to be a big sister. But I feel like I’m mourning something. I feel like the relationship that I have with my girl is going to change. I have a fear that I might not love the baby the way I love her. Pleaseeee don’t judge or bash me. I talked to my sister and she told me she had the same feelings with her second baby. Does anyone else feel this way? Am I the only one with these crazy feelings?*

Because these were pregnant mothers, not surprisingly, a good deal of the posts and LG’s focused on pregnancy-related issues. Mothers discussed normal prenatal check-ups at the doctor’s office (“*tomorrow is my blood work*…*in about a week I will know the sex of my baby!”*) and provided suggestions to others for early pregnancy symptom management, such as nausea or leaky breasts *(“had dry mouth 1 day last week.the [nurse] said it was likely just hormones from an increase in progesterone”*). Prior to their ultrasounds to reveal the sex of the new baby, mothers often tried to guess the baby’s sex and debated about possible early sex-detection procedures.

After mothers had found out the sex of the baby, they discussed the ultrasound experience and conversed about their feelings about having a boy or girl. They often announced their child’s sex to the BabyCenter community and mentioned how they planned to share the news with family and friends. Not surprisingly, possible baby names were a source of great discussion. Community members offered suggestions for possible names, with the decision of the chosen baby’s name ultimately announced to the community. As one mother posted:

*we are having a hard time thinking of baby names! We like Katy for a girl but can’t think of a middle name. We don’t have a clue for a boy name LOL. Any suggestions?*

Other topics of enormous discussion were their diet and their weight gain in comparison with their first pregnancy, with many women stating they often gained more weight with their second baby than they did with their first (*“I am eating what I want*…*.I’d rather not stress about meal plans and food*……*It didn’t take me long to get all the weight off after my son was born so I’m not concerned”*). They also discussed what it was like to see the ultrasound images for the first time, to see and hear their babies’ heartbeats, and often shared prenatal test results and celebrated or grieved the outcomes with the BabyCenter community. The following post by a worried mother underscores this desire for support:

*My blood screening test results came back. The doctor said my baby’s spinal cord and brain development number is abnormal.*……*.I’m so worried that I’m going to see a maternal-fetal medicine specialist next week to find out what’s going on. Has this happened to any of you?*

As their pregnancy progressed, mothers continued to turn to the BabyCenter community for advice, support, and guidance. Mothers discussed their firstborn children’s evolving roles and adjustment and traded advice on how to prepare their children for a new baby brother or sister. Mothers having already made the transition with a newborn at home would often share stories of how their children were adjusting to their new place in the family system and what they had done to support their children. Here is what one mother posted about announcing she was pregnant to her firstborn:

*Well, we told our 3 year old today that he is going to be a big brother. We showed him a picture of the ultrasound, got him a nice book about being a big brother and let him ask any questions he wanted. He was curious at first and asked questions (where is the baby, how big and why is she in mommy’s tummy)*…*half an hour later, he started hitting me saying he wants to squash the baby*…*Then his dad went and talked to him and he said he was frustrated and jealous—he took his ‘the way I feel’ book and showed how he was feeling and that he was afraid that we won’t take him to the pool or play with him anymore.*

Mothers also posted about social support they received from two separate communities, including their daily, real-life support system of family and friends and the online support of the BabyCenter community *(“Pregnancy seems to be one worry after another*….*guess there is really no way of knowing if this will affect the baby. But this is on my mind and you are all my best support. Thanks for reading.”*). With respect to their real-life support system, mothers discussed how members of their families or friends were either there or not to meet their emotional needs. They also discussed their needs for assistance as the end of the pregnancy approached and labor and delivery were imminent. With respect to the BabyCenter community, mothers often shared humor with each other, telling funny anecdotes, but also asking for very specific advice about pregnancy or baby-related topics and deliberated over and shared their baby-related material wish lists and where to purchase those items *(“I need stuff as small as bottle brushes, pacifiers, and nipples for bottles*…*.I’ll always need diapers, wipes, lotions, shampoos, and clothes”*).

As mothers moved into later pregnancy, discussions turned to topics relevant to their approaching due dates. Stay-at-home mothers discussed the added pregnancy-related financial stress of covering the needs of two children and their expanding family system, whereas working mothers talked about timing their maternity leaves, how tricky it was at times to manage their paid time around labor and delivery, and debated the pros and cons of daycare. One mother talked poignantly about the challenge of managing work and time off for the birth of her baby:

*Unfortunately, I’m working RIGHT up until birth because I haven’t been at my job long enough to qualify for FMLA [Family Medical Leave Act] so I have to use all my sick days to get any time off. I’m working a half day and then going in for a C-section the next.But it totally SUCKS! I wish I could take some time off to get through these last super uncomfortable weeks and finish doing all the things at home to feel 100% ready.*

As pregnancy progressed, mothers discussed late pregnancy-related discomfort, such as cramping or early Braxton-Hicks contractions. As would be expected, there was considerable discussion of topics related to preparing for labor and delivery, where they had decided to labor and give birth (e.g., hospital-birth or home-birth), whether they were choosing a vaginal birth after C-section or not, and if the latter, how they were preparing for birth by C-section and choosing the dates for the scheduled C-section. As their due dates drew closer, many mothers discussed specific birth plans and preparations for labor and delivery, often discussing who would accompany them during the delivery and who would be responsible for caring for the firstborn children. The following post exemplifies some of these discussions:

*My 1st daughter was a breech baby. We decided to schedule a c-section. I had zero complications and had an amazing experience. I was nervous but it turned out great*……. *This time, we are doing a vbac [vaginal birth after Cesarean]*

### Timeline for Post-birth Topics Posted by Second-Time Mothers

Supplementary Table 2 summarizes the LG’s and labels for the 20 topics gleaned in the post-birth LDA, and we provide here examples and quotes of some of the posts representative of those LG topics. Recall that the LGs generated are based on a temporal post-birth timeline. Because the post-birth data started with the month of the due date, most LGs near the beginning of this dataset concentrated on the experience and aftermath of labor and delivery. Mothers often turned to the BabyCenter community to share early labor symptoms and seek advice about whether *“it was the real deal.”* One topic focused specifically on carpal tunnel syndrome, a common ailment in pregnancy when there is a build-up of fluid in the tissues of the wrist and mothers with such a condition solicited advice for symptom relief. Mothers also related their concerns regarding labor and delivery and the logistics of managing delivery and family arrangements, particularly with respect to how to care for the firstborn while they were in the hospital:

*DH [dear husband] will be with me at the hospital for the first night while his mom is with DD [dear daughter]. I’ll spend another day at the hospital with DS [dear son] alone after DH goes home.*

After the birth, mothers discussed their general labor and delivery experiences. Mothers often gave labor and delivery timelines and detailed their birth story to the group, as exemplified by the post of this mother:

*Started Pitocin at 7 a.m. and hard contractions started almost immediately. Got epidural (pure heaven), water broke on its own and I was at 10 cm and ready to push.she was born*….*No tearing/cuts, fast and easy, felt no pain. It was amazing. We left hospital after 24 h and we’re happy to be home with big brother.*

Mothers also discussed general recovery after birth and specific to recovery from a C-section, but also now commenting on what it meant trying to do so with two children at home (“*I’m terrified of my C section recovery on top of caring for a newborn and toddler”*). Mothers sought advice for recovery tips, commiserated over postpartum pain, and discussed pain medications. During this time, mothers also shared announcements of their baby’s birth and celebrated with the BabyCenter Community *(“Congratulations on your new precious bundle of joy”*). Finally, they discussed the baby’s health shortly after birth, such as temperature, flu symptoms, or test results.

Because there was a new baby in the home, mothers naturally talked about feeding, by both breast and bottle, and discussed the joys and issues surrounding their new baby’s eating habits. Conversations ranged from milk production to baby’s hunger signals, or advice on supplementing breastmilk with formula *(“you can supplement with formula but if you don’t need to*….*then introducing formula may end up hurting your milk supply”*). Once home, mothers shared stories of how the first few weeks were going for their family and provided suggestions to help with the transition. During this time, mothers focused on the new baby and the family members’ reactions to the changes, as this mother posted:

*in these early weeks things can all become a blur*…. *It can be a rough time for parents thinking things might be wrong or they might be doing something wrong. But in reality it’s just an intense period of growth and development for your LO [Little One]. She’s so adorable DH [dear husband] and I can’t stop staring at her. Son is overwhelmed.*

Mothers also discussed their family’s adjustment to the addition of a new baby and shared stories of their firstborn children’s reactions to their new role (both positive and negative) as an older sibling, communicated the difficulties inherent in trying to maintain a relationship with their partner during this demanding transition, and worried about their ability to care for two children at different developmental stages. Here is one mother’s description of the complicated balance between the loving acceptance of the baby by the firstborn and dealing with these children’s challenging behaviors:

*Since being home my DD [dear daughter] LOVES her new little sister and wants to help and do everything for her. She is so sweet (holding her hands, saying I love your beautiful fingers, I love your beautiful hair LOL) BUT she is acting out too. She was the center of our universe before LO [Little One] and now obviously has to share the spotlight. She is back-tracking with toilet training and purposely going in her pants. She is also running around and being super-hyper and won’t listen. If I warn her about a punishment that’s coming she will make sure to do it again like she wants to be punished to get the extra attention. She also keeps taking LO binkies and crawling around acting like a baby. I am trying to handle it with patience and sitting her down and explaining that we still love her and making sure to give her one on one time but I can feel my patience thinning and with all these hormones from birth I know I’m going to end up snapping. Any advise is welcome! I want my sweet toddler back!*

Relatedly, mothers discussed fears such as getting two children in and out of the car or dealing with toddler tantrums while caring for a baby. They sought advice from each other regarding strategies around caring for two children once their support network of family and friends stopped visiting and the mother became the primary caregiver.

*I don’t know what I’ll do after next week when I’m on my own dealing with a newborn and a toddler that screams constantly, won’t nap without 2 h of cuddling, and then turns bedtime into a marathon nightmare scream-fest. Anyone else with a young toddler of the needy variety have advice to offer?*

Finally, mothers discussed how to arrange life with two children. Though similar to their concerns over their ability to care for two children, this topic focused more on the physical modifications needed, such as changing room arrangements for both the older sibling and new baby, and switching from a crib to a toddler bed for the now older sibling *(“We kept our son in our room in his own crib at first to make things easier at night*….*moved him when he was 8 months old*….*Now both boys have to share the room”*).

As time progressed, mothers expressed concern over *“what was normal,”* which ranged from unpleasant smelling umbilical cords to excessive spitting up after feeding. Depending on the issue, mothers in the community generally reassured the worried poster or suggested they seek medical advice. They also talked about the new baby’s sleep habits and traded suggestions for getting a baby to nap while other mothers discussed sleep schedules *(“Mine rarely naps for more than 30 min at a time and he’s also 5 months old”*). Similarly, there were discussions about the new baby’s feeding and bowel habits, such as the optimal spacing between feeds and what was normal with respect to bowel movements. Many mothers discussed their new baby’s health more generally, often worrying about their baby’s acid reflux or indigestion issues. As the weeks progressed, their posts focused on work-family balance as parental leave ended and mothers started to return to work, with mothers either celebrating or commiserating the return to “normal” family routines and going back to work after maternity leave. Here is one mother’s post:

*Anyone else going back to work this week? I forgot how hard this transition is. I cried a lot this week! I wish there was more time, but I’m grateful for every minute of my maternity leave with my little guy.*

## Discussion

The main purpose of the present study was to isolate both pre- and post-birth topics using text from posts on the parenting website BabyCenter LLC by second-time mothers in order to determine what these women discussed in their online communities and whether there were specific concerns or anxieties arising for these women. The earlier qualitative work based either on single interviews with one mother or a small sample of mothers suggested that second-time mothers often voiced some of the same concerns as first-time mothers with respect to pregnancy, prenatal testing, choosing a baby name, the gender reveal, and the overall health of the baby during the early months of life. Yet, second-time mothers also expressed unique concerns, particularly around their new role as the mother of two young children, the ability to care for both children, and whether they could love another child as much as they loved the first. Given the paucity of research on second-time mothers and the frequent focus on first-time mothers in the perinatal period, the current study offered a unique perspective into the on-line world of a group of women expecting their second baby and what it meant to become a mother of two children. Given the availability of online resources and the time that has elapsed since the earlier studies, one might think that these women have access to an abundance of resources and information to help alleviate any worries about being the mother of two children. But, as we will discuss, this did not appear to be the case. Thus, there is a need by researchers and practitioners to attend to the varied needs of women as they approach pregnancy and parenting for the first, second, or even later times over their life course. Believing that once a woman has given birth the first-time, she knows all there is about motherhood may be an oversight in providing care and support for second-time mothers (Mercer, 1979). The following discussion will highlight some of the key topics of second-time motherhood discussed in the BabyCenter birth clubs along with the implications of this work for practitioners and researchers.

### Pregnancy and Postpartum Concerns of Second-Time Mothers

Because the dataset was gleaned from BabyCenter LLC birth clubs, most of the identified LG’s reflected mothers’ feelings and thoughts about their second pregnancies and the impending changes to family life with the birth of their second child. Mothers in the current study overwhelmingly discussed their experiences with pregnancy and the postpartum period and did so in great detail. Mothers’ posts targeted both typical (e.g., what type of foods alleviated morning sickness) and more serious issues (e.g., worry over the health and safety of the unborn baby), and spanned the entire time frame from pregnancy to post-birth encompassed by the current dataset. Taken together, these findings suggest that second-time mothers did not appear to effortlessly navigate their pregnancies, deliveries, or recoveries with no worries or concerns simply because they had already experienced one pregnancy and the birth of their first child. Instead, these mothers focused on a variety of pregnancy symptoms and were continually concerned about their pre- and post-natal experiences, as many pregnant women no doubt are. Thus, the findings indicate that second-time mothers realize that each pregnancy is different, and therefore, need as much support and assistance through their second pregnancies by both healthcare practitioners, friends, and their family as first-time pregnant women.

### Will I Love My Second Baby as Much as My First?

Another topic conveyed by second-time mothers was a concern that they may not be able to love their second baby as much as their first, a topic also mentioned in the earlier qualitative work, but which is given very little attention in current research and practice (Jenkins, 1976; Mercer, 1979; Walz and Rich, 1983). No research to date has attempted to assess mothers’ attachment to a second baby with another child in mind. Popular media confirms that this is a topic on the minds of second-time mothers as evinced by a segment of the popular morning show, *Good Morning America*, that aired on January 26, 2018. This episode addressed the anxieties of their ABC News chief meteorologist, Ginger Zee, as she prepared for the birth of her second baby (Sherwood, 2018). Zee posted a picture of her firstborn and asked her Facebook audience “how will I ever love the second one as much as I love this little boy?” The question garnered over 5,000 responses in a 24-h period with many mothers sharing similar thoughts on the site. For instance, one woman wrote *”I had the same fear, but that went away as soon as I held my second child. Your heart grows bigger and makes as special a place as your first one is in.”* Another wrote: *”I remember thinking the exact same thing, I cried the final two weeks thinking I’d have to split me in two to show them both love and attention. BUT! As usual. women are amazing beings. Loving two the same comes naturally. You’ll see!”*

Similarly, in an article published in the Huffington Post, Shapiro (2014, updated 2017) documented a similar struggle, stating that she truly believed there was no way she could love her second child as much as her first.

*When I was pregnant with my second child, my husband and I affectionately (and jokingly) began to refer to him as “Baby Chopped Liver.” You see, we already had our firstborn – our golden child, our prince, our special, special boy. And though I wouldn’t have admitted it at the time, while pregnant, I thought to myself often that I would always do my best to make Baby Chopped Liver (hereinafter referred to as “Little BCL”) feel like he was loved just as much as his brother, even though it obviously could never be true. In my ninth month of pregnancy, I sat on the floor of my son’s bedroom and cried as I read to him, mourning that our time alone together was coming to a close. I might have even resented the fact that, despite all of my wishes for a second child, there was going to be another human being who would need me and detract my attention from my perfect son. I thought, how on earth could I possibly love anyone or anything as much as my firstborn?*

Shapiro goes on to describe her feelings once her second baby was born:

*And then Little BCL was born. They placed him in my arms and he was tiny and perfect. And he looked up at me and gave me that look – you know the one. He opened up his eyes and looked straight into mine and I could almost hear him say, “Hi Mom, I’ve been waiting to meet you.” And months down the line, when my firstborn was throwing tantrums and picking his nose with abandon, Little BCL was napping and cuddling and cooing and smelling like all of those wonderful baby smells you forget about once you have dirty toddler diapers and dirty toddler hands to battle.*……. *So, the impossible did happen. I loved Little BCL as much as my firstborn – as much as anything in the world. All it took was meeting him for the first time.*

Based on popular media, past interviews with second-time mothers-to-be, and the findings of the current study, some second-time mothers appear to have serious concerns about their ability to love a second child as much as the first, but how many mothers actually feel this way and do these anxieties generally dispel after the birth of their second child? We simply do not know. There is insufficient empirical research on mothers with two or more children to know how frequently women worry about loving two children at once when expecting their second baby and hence, what the time course is for these worrisome thoughts. Prior research on maternal-fetal attachment has found that perseverative thinking (e.g., rumination and worrying) during early pregnancy predicted impairments in maternal-fetal attachment and increased depressive symptoms in late pregnancy (Schmidt et al., 2016). Further, de Cock et al. (2016) reported that mothers’ difficulties in bonding with the baby during pregnancy were associated with increased anxiety and parenting stress, and less partner support, Moreover, poor maternal-fetal bonding during pregnancy continued to predict poor bonding into toddlerhood. Thus, these feelings may not subside in some cases and may have repercussions for the developing mother-infant relationship and children’s social and emotional development (Branjerdporn et al., 2017). Without further research, it is difficult to know if, and when, we should be concerned if these feelings continue into the postpartum period for second-time mothers. If mothers are finding it difficult to bond with their baby over time, it may be necessary to assist women struggling emotionally with these thoughts in the last weeks of pregnancy, rather than assuming such thoughts are “normal” for mothers having a second baby. In sum, because this topic was a cause for concern for mothers in the early research decades ago and continues to be raised on both parenting websites and popular media, we advise both researchers and health care workers to attend to these concerns seriously in order to elucidate whether we should provide support and services to these women and their babies.

### The Importance of Social Support During Second-Time Motherhood

Another issue voiced by mothers on BabyCenter LLC in the current study was their desire for social support, particularly from their partners and extended family and friends. LGs mentioning social support were also prevalent, indicating that mothers often posted about family and their social networks. Second-time mothers sought emotional and material support from their real-life social network, but they also reported feeling forgotten and treated by others as if having the first baby prepared them for all there was to know about motherhood—“she knows the ropes” (Mercer, 1979). Unlike earlier generations of mothers who may have felt isolated and alone with these thoughts, mothers in the current study leveraged support from their online BabyCenter community. Second-time mothers turned to others for emotional support when they experienced frustration, sadness, joy, or excitement. Along with findings from previous research on parents’ online behavior (Sarkadi and Bremberg, 2004; Dworkin et al., 2013), the current findings indicated that the online world may provide mothers the opportunity to candidly discuss their worries and fears (Schoenebeck, 2013), while also offering settings to receive targeted support from a large and diverse network.

### Seeking Advice Online About Pregnancy, Parenting, and Infant Health

Probably the most unique characteristic of mothers in the current study compared to earlier generations before the advent of the internet is the sheer availability of internet websites such as BabyCenter LLC to seek advice about adding a new baby to the family. These mothers were able to connect with thousands of other potentially geographically dispersed mothers in a similar situation and had the opportunity for many interactions in which they sought or shared guidance on their pregnancies, the impending transition with a second baby, and other baby-related topics. As such, these findings indicate that online communities may be an advantageous target for interventions that seek to distribute factual and empirically based information about the transition to the second child.

Moreover, current research suggests that while some firstborn children experience disruption and an increase in problem behaviors, many others often respond positively to the birth of their infant sibling (Oh et al., 2015; Song and Volling, 2015) or experience little to no distress (Volling, 2012). Even so, there is still plenty of misinformation available online surrounding the birth of a second baby giving the impression that the experience is universally disruptive and potentially traumatic for young children (e.g., Boyd, 2009; Cooper-Abbs, 2013; Dais, 2016). The individual variation in children’s adjustment following the birth of a second child (Volling, 2012) would suggest that while some mothers’ concerns may be well-founded, others may be unnecessarily anxious about the transition due to misinformation being shared on internet sites that does not accurately reflect current research on the transition to siblinghood (see Volling et al., 2017). Taken together, these findings suggest that second-time motherhood is a transition unique from first-time motherhood. By isolating the topics mothers posted and discussed in their online BabyCenter community, the current study provided some initial insights into how to assist and support women as they transition from a mother of one to the mother of two children.

### Strengths and Limitations

One of the strengths of the current study was its use of the pseudonymous forums for second-time mothers on BabyCenter LLC, which provided a data-rich contemporary arena to investigate the posts of second-time mothers. The BabyCenter birth clubs seemed to provide a largely supportive outlet in which mothers could openly discuss their feelings about pregnancy, birth, and parenting, with fewer societal pressures than they might encounter in face-to-face contexts. Utilizing data from BabyCenter LLC, the current research allowed us to obtain textual information from a large sample of approximately 4,000 unique posts of English-speaking mothers expecting and eventually giving birth to their second child, who appeared to be largely located in the United States. As such, our results can be generalized to a larger population of second-time mothers than earlier qualitative studies that interviewed only a single woman or a few women, several of whom were reporting parenting difficulties. The current results may provide a more uninhibited picture of mothers’ thoughts and feelings during second-time parenting.

Because the project needed to be completed within a relatively short time frame (Beyers-Carlson, 2018), one of the limitations is that we were only able to sample posts across two birth clubs spanning 2016–2017. Further, the posts were from mothers predominantly residing in the U.S. Additional BabyCenter sites are now available in 11 different countries (e.g., Canada, Australia, Brazil, Germany, France, India) and as of this writing, company information on its website states that “BabyCenter is the world’s number one digital parenting resource….Around the globe, BabyCenter is available in nine different languages and more than 50 million parents visit BabyCenter’s 11 websites every month” (BabyCenter, LLC, n.d.; see text footnote 2). Mothers in other countries may discuss different topics relevant to cultural practices concerning pregnancy, birth, and child-rearing that were not the focus here and further research is needed to determine what topics women of other cultures discuss as they approach second-time motherhood. The point to be underscored here is that forums such as BabyCenter LLC are now used worldwide by mothers for both advice and support on pregnancy, parenting, and infant development so these on-line forums are a significant source of information (or misinformation) for women.

Another limitation is that we restricted the LDA models to generate no more than 20 LG’s at both pre-birth and post-birth so it is certainly possible that there are additional topics that have gone undocumented here. This decision was made given the exploratory nature of the research and the fact that few studies actually consider the concerns of second-time mothers; we wanted to limit our focus to the most frequently discussed topics. Although BabyCenter LLC does have father-specific Groups (e.g., Dads Only), most active users in the second-time parenting birth clubs appeared to be mothers, evidenced by the overwhelming focus on maternal experiences (e.g., pregnancy, breast-feeding). Clearly, fathers must also adjust to the changing role of father of one to father of two and may also desire information on the transition. Fathers are also turning to the internet for information (see Fatherly, n.d.)^3^ and further research is needed to uncover what men may discuss or seek advice for when they are approaching second-time fatherhood. In sum, discussion boards and posts left by parents on these sites offer a rich opportunity for researchers to move out of the lab and into the on-line lives of parents and their communities of support.

The overwhelming majority of conversations about partner relationships appeared to be written by female partners within heterosexual relationships, as evidenced by the repeated use of DH (dear husband), and it is unclear whether same-sex couples also use these boards to discuss these issues. Finally, though the anonymous nature of online message boards can provide safe environments, the environment of anonymity and disinhibition may also lead to performative posts in which users make comments or express ideas in order to gain attention, likes, or other reactions from users in the group.

## Conclusion

In conclusion, most women have more than one child and the results here suggest that second-time mothers are discussing and asking for advice on several topics not voiced by first-time mothers such as their ability to love the second baby as much as their first child and the logistics of caring for two children. Yet, few parenting resources are geared specifically to the needs of these mothers, leaving them to turn to online communities for knowledge and support. There is a clear need to address the concerns and anxieties of these mothers, given that some of their anxieties may actually be perpetuated by misinformation on the transition period described on many websites, including professional websites, as a time of disruption and even crisis for firstborn children. Recent research suggests that most children experience a minor period of adjustment in the first months after birth but adapt shortly thereafter (Volling et al., 2017). Parents (both mothers and fathers) are able to maintain strong coparenting relationships after the birth of a second baby (Kuo et al., 2017) and in some instances, the mother-child and father-child relationships actually improve after the birth of the second baby (Volling et al., 2021). On-line communities may present an efficient avenue for providing mothers and fathers with factual information to help ease any anxieties about the transition to second-time parenthood and offer parents the support necessary to become a family of four.

## Data Availability Statement

The raw data supporting the conclusions of this article will be made available by the authors upon reasonable request.

## Ethics Statement

Ethical review and approval was not required for the study on human participants in accordance with the local legislation and institutional requirements. Written informed consent for participation was not required for this study in accordance with the national legislation and the institutional requirements.

## Author Contributions

EB-C, SS, and BV conceptualized the main ideas for the manuscript. EB-C conducted the main analyses, reviewed the results, interpreted the results, and wrote the first draft of the manuscript. SS and BV reviewed the results, provided support for interpreting the results, and assisted with writing the manuscript. All authors reviewed and approved the final submitted version.

## Conflict of Interest

The authors declare that the research was conducted in the absence of any commercial or financial relationships that could be construed as a potential conflict of interest.

## Publisher’s Note

All claims expressed in this article are solely those of the authors and do not necessarily represent those of their affiliated organizations, or those of the publisher, the editors and the reviewers. Any product that may be evaluated in this article, or claim that may be made by its manufacturer, is not guaranteed or endorsed by the publisher.

## Footnotes

1
babycenter.com

2
https://www.babycenter.com/about

3
https://www.fatherly.com/
