# Supplementary material for: Mother of One to Mother of Two: A Textual Analysis of Second-Time Mothers’ Posts on the BabyCenter LLC Website
Source: Front Psychol. 2022 Apr 25;13:859085. doi: 10.3389/fpsyg.2022.859085 (PMC9082668; doi:10.3389/fpsyg.2022.859085)
Supplement: Supplementary file 1 [file Table_1.docx]

**Table S1**

***Topics from the Pregnancy and Pre-Birth LDA Model for Second-Time Parenting Groups***

| **Lexical Group and Topic Labels** | **Example Post** |
| --- | --- |
| **Sharing pregnancy announcement with friends and family** peopl,post,famy,com,group,bump,pic,friend,us,say,tel,shar,ask,know,everyon,see,look,moth,law,col,pleas,annount,if,her,on,mil,pict,let,lov,interes | "W*e wanted to do a fun themed announcement…to do something BIG because people are certainly going to be surprised cuz we've insisted from day one that we weren’t having anymore kids."* |
| **Safety concerns** ye,tak,vitamin,b,ut,temp,much,preggo,bath,warm,smok,doc,tummy,  okay,said,artic,outsid,gummy,fol,reach,intercourse,dress,insid,uter,  rol,swe,nest,dai,warn,the | *"So just found out that we're pregnant yesterday. I'm researching vitamins and can't decide which one. I took the Nature brand with DD (dear daughter) 4 years ago, and took them again months ago until I ran out. We were ttcing [trying to conceive] during that time but I gave up stressing and forgot to get more vitamins."* |
| **Can I love my second child as much as I love my first?** i,pregn,feel,week,baby,lik,know,first,tim,think,on,real,get,so,want,thi,second,last,much,worry,ev,sint,show,felt,but,day,lol,start,going,ho | *“Hello everyone, I'm a little conflicted about the way I feel about this pregnancy. Me and my husband have a 5 year old wonderful girl. I just finished school and we planned to get pregnant right after I graduated. That was in May. Being 26 I got pregnant the first try. I just found out on Sunday and I got very nervous. I thought I was going to react differently, be over the moon happy. But when I found out I felt happy but also a little sad...maybe. I mean, we had been planning this for a long time and my girl is excited to be a big sister. But I feel like I'm mourning something. I feel like the relationship that I have with my girl is going to change. I have a fear that I might not love the baby the way I love her. Pleaseeee don't judge or bash me. I talked to my sister and she told me she had the same feelings with her second baby. Does anyone else feel this way? Am I alone with these crazy feelings??* |
| **Normal prenatal check-ups at doctor's office** im,u,dont,confirm,hav,cant,me,didnt,that,hair,yet,r,bean,puk,back,st,  h,tho,bil,bloodwork,transvagin,uns,serv,friday,pres,box,green,very,  ramz,isn | *"Had my sizing u/s [ultrasound]today. On track! Baby was dancing and had a HR of 171. Yay! Tomorrow is my bloodwork including panorama test and in about a week I will know the sex of my baby!”* |
| **Managing early pregnancy symptoms (e.g., nausea, leaky breasts)** progesteron,milk,ad,effect,produc,fruit,breast,secret,com,staff,leak,  babyc,peppermint,liquid,answ,you,keep,accid,fee,zero,org,mass,mad,perhap,o,strange,can,pedy,gum,zi | *“I had dry mouth one day last week. I wasn't thirsty or dehydrated. Water didn't seem to help. It only lasted a day. I mentioned it to my nurse yesterday and she said it was likely just hormones from an increase in progesterone, just a random pregnancy symptom...since it just lasted a day. But, it was super annoying.”* |
| **Morning sickness and sleep changes** i,day,week,pregn,feel,tim,sick,first,last,get,lik,morn,night,tak,nause,  symptom,every,today,it,my,start,stil,ev,tir,on,norm,thi,bad,sleep,nau | *"This pregnancy feels so much harder! I've experienced the same amount of morning sickness as the last one...which is all day. Luckily, that has subsided a bit the past few days. The thing that is different is the level of exhaustion."* |
| **Baby's sex** i,week,baby,ultrasound,boy,girl,see,due,th,dat,day,said,wait,today,  think,first,ear,gend,meas,lin,my,scan,find,we,the,look,hop,anoth,  would,doc | *"Went for U/S (ultrasound) at 23 weeks just to check gender and was told it was a boy. Then had to be checked again at 26 weeks cuz my amniotic fluid is low, a different U/S tech was sure its a girl. DH [dear husband] decided to get a second opinion from another tech who said he was 99% certain that it is a girl. Has this happened to anyone else?"* |
| **Diet** i,eat,drink,try,help,wat,pregn,food,tak,keep,it,mak,us,lot,also,tri,ev,  control,conceiv,much,good,work,sur,hormon,crav,sug,know,anyth,  meal,wan | *"I am eating what I want. Even if I eat unhealthy stuff after a day or two I end up wanting healthy food so it balances out. My portions are smaller because baby is pressing on my stomach so I can't load up on bad stuff. I’d rather not stress about meal plans and food organization and get hit with a craving. I'm saving those for after the baby. It didn't take me long to get all the weight off after my son was born so I'm not really concerned. …. Kind of did a veggie bowl but I just wanted the chips and guac really been craving guac like crazy// Hey, I've been eating a lot of fruit lately which is good . I hope I don't start craving junk food later on. My biggest craving with my first one was peanut butter. So I ate anything with peanut butter, pb cups, butter finger, sometimes just a spoon and the jar lol. I don't even like peanut butter. What was your biggest craving?* |
| **Pregnancy weight gain in comparison to first** anyon,els,pregn,i,expery,has,week,weight,level,gain,hcg,lbs,doe,first,ear,bfp,dark,er,cury,lost,my,healthy,pound,fat,today,got,worry,big,  just,d | *"My weight has also increased.. I’m almost at the weight that I was when nine months pregnant.. So maybe this is also a cause.."* |
| **Baby names** nam,lik,lov,girl,boy,i,we,fatigu,middl,pick,cut,preg,decid,eg,fing,  cross,yog,hah,calc,it,think,sound,or,our,pop,receiv,april,wrap,acn,loo | *"We are having a hard time thinking of baby names! We like Katy for a girl but can't think of a middle name. We don't have a clue for a boy name LOL. Any suggestions? I'm pretty sure we're having a girl."* |
| **Concerns about pregnancy and heartbeat** sorry,heartb,heart,norm,hear,sex,no,baby,heard,cervix,fin,sam,is,beat,min,that,cup,doct,ok,said,round,sound,check,everyth,braxton,littl,  coff,sign,hick,su | *"I went to the dr and they did a cervix check at 12 weeks and it was closed up tight. No blood. She cleaned me up, but I still had discharge regularly for about a month. I attributed it to constipation because I couldn't figure out what else it could be.* |
| **Test results and outcomes for maternal and baby health concerns** test,i,posit,blood,took,day,march,period,got,high,neg,back,said,lat,  result,yesterday,anoth,cam,see,they,doct,pregn,miscarry,on,went,tak,due,would,follow,uri | *"My blood screening test results came back. The doctor said my baby's spinal cord and brain development number is abnormal …. I'm so worried that I'm going to see a maternal- fetal medicine specialist next week to find out what's going on. Has this happened to any of you? Thank you for any comments and support!!"* |
| **Older sibling adjustment** i,old,baby,my,year,husband,want,dear,son,daught,we,tim,get,on,he,  month,kid,littl,mom,going,she,dd,famy,room,dh,new,us,know,big,li | *“Well, we told our 3 year old today that he is going to be a big brother... We showed him a picture of the ultrasound, got him a nice book about being a big brother and let him ask any questions he wanted. He was curious at first and asked questions (where is the baby, how big and why is she in mommy's tummy)…half an hour later, he started hitting me saying he wants to squash the baby…Then his dad went and talked to him and he said he was frustrated and jealous - he took his ‘the way I feel’ book and showed how he was feeling and that he was afraid that we won't take him to the pool or play with him. anymore”* |
| **Seeking support** would,baby,if,i,it,nee,may,get,lot,also,you,thing,saf,help,they,tak,  find,pregn,the,support,high,good,might,put,much,wom,lik,matern,  bag,fa | “*Pregnancy seems to be one worry after another. Has anyone had a similar experience but everything was okay? I guess there is really no way of knowing if this will affect the baby. But this is on my mind and you are all my best support. Thanks for reading.”* |
| **Receiving support** good,luck,pray,congr,wish,congrat,best,hop,success,mam,welcom,ur,send,abdomin,wel,was,healthy,mama,look,determin,sweet,fun,al,way,reply,perfect,heal,it,ish,i | *“Congratulations and welcome!! I wish you a happy, healthy pregnancy.”* |
| **Seeking and giving advice** thank,any,adv,lady,hi,year,help,n,expery,apprecy,pleas,anyon,new,  twin,wond,old,how,ide,gre,what,board,hey,mom,suggest,thought,  baby,tip,gift,guy,man | *"I had to use a pump to establish milk supply. Formula saved my twins, and honestly I never made enough for both so I chose to supplement with formula long term. You are doing great by providing milk. Do what works best for you."* |
| **New baby purchases** i,us,on,buy,seat,siz,baby,bought,nurs,car,cloth,lov,smel,com,wear,fit,diap,brand,stor,got,sensit,ov,fre,new,opt,also,doubl,bra,stroller,stuf | *" still using things such as fleece blankets, his crib (we have a convertible crib), and dresser. I need stuff as small as bottle brushes, pacifiers, and nipples for bottles. I also donated some stuff to a family, and gave up things I didn't love from my first (my diaper bag and changing table). Also, I'll always need diapers, wipes, lotions, shampoos, and clothes."* |
| **Work and family balance** i,work,get,would,nee,tak,lik,tim,us,mak,car,you,help,go,know,hom,  job,it,want,good,if,thing,ins,try,real,think,on,ev,leav,ne | *“Unfortunately, I'm working RIGHT up until birth because I haven't been at my job long enough to qualify for FMLA [Family medical Leave Act] so I have to use all my sick days to get any time off. I'm working a half day and then going in for a C-section the next...But it totally SUCKS! I wish I could take some time off to get through these last super uncomfortable weeks and finish doing all the things at home to feel 100% ready.”* |
| **Pregnancy discomfort** i,pain,cramp,blee,back,lik,low,it,get,sid,spot,press,feel,period,walk,  norm,help,hurt,sometim,bad,sit,left,lay,lot,head,right,heavy,us,caus,le | *"I've been having contractions that I have felt since yesterday morning. All over the place on time and pain (stomach, back, pelvic pressure, cervical pain) so unless my water breaks between now and Thursday afternoon, I'll see what dr says at my appt. I'm thinking she is going to send me to l&d [labor and delivery] or tell me to stop the meds."* |
| **Labor and delivery preparation (e.g., C-section, vaginal birth)** i,week,doct,lab,c,hospit,sect,my,baby,bir,go,first,said,tim,cal,ob,  would,going,went,contract,dr,check,get,vagin,told,hour,appoint,last,  expery,de | "*My 1st daughter was a breech baby. We decided to schedule a c-section. I had zero complications and had an amazing experience. I was nervous but it turned out great. The down side was the long hospital stay and about a week recovery but overall an amazing experience. This time, we are doing a vbac [vaginal birth after Cesarean."* |

*Note.* The label for the LG topic was generated through consensus and is displayed along with tokenized keywords from the LG’s resulting from the LDA model. LG’s are listed in temporal sequence starting with posts early in pregnancy and ending with posts in late pregnancy. To protect the privacy of individual posters, each LG includes a lightly anonymized quote from a post as an example.

**Table S2**

***Topics from the Post-Birth LDA Model for Second-Time Parenting Groups***

| **Lexical Group and Topic Labels** | **Example Post** |
| --- | --- |
| **Health concerns (e.g., carpal tunnel, acne)** ongrat,precy,midw,turn,girl,rec,so,may,ther,pretty,complet,anyth,oft,  said,littl,fin,needy,hir,skin,much,also,pea,tunnel,leaflet,defo,carp,far,  good,talk,alar | *"I have the same issue - it's carpal tunnel - look online for some exercises. Ask your dr for a pamphlet but definitely call her and let her know too."* |
| **Specific labor and delivery logistics** nee,good,feel,dont,tak,baby,want,famy,new,luck,you,right,do,tim,heart,situ,it,thing,pregn,many,tel,back,friend,expery,go,alon,rel,hop,delivery,fin | *"DH [dear husband] will be with me at the hospital for the first night while his mom is with DD [dear daughter]. I'll spend another day at the hospital with DS [dear son] alone after DH goes home."* |
| **Concerns regarding labor, delivery, and recovery** march,th,due,baby,dat,scheduled,son,ds,min,nam,cam,dear,ar,week,nd,mild,ear,born,induc,oz,sect,my,hour,what,c,soon,contract,tim,so,wa | *"I'm terrified of my C section recovery on top of caring for a newborn and a toddler."* |
| **Decisions around delivery** i,baby,induc,com,would,girl,hand,my,thank,stil,week,think,told,due,  see,on,tak,way,sur,it,opt,ev,delivery,daught,right,long,thing,and,try,posi | *"I’m 3 days overdue and induction is set for next week. I was not induced with my first born. I'm a little curious about induction. Anyone been induced before and can tell me what it is like? Should I be worried or scared?"* |
| **Early labor symptoms** muc,plug,week,lost,i,sound,day,today,ago,could,lab,los,grow,two,stil,  induc,go,but,drop,show,hap,nos,thi,spurt,mean,posit,big,u,bloody,preg | "*Well ladies what can ya tell me about these. I had 2nd sweep 2day and my mucus plug is coming away. My back is getting very sore and getting pressure, pain is coming and going, also pressure near back passage. When do u know if it's the real thing or not?"* |
| **Labor and delivery** i,hour,week,contract,lab,tim,day,start,baby,hop,wat,induc,went,first,my,brok,get,night,feel,today,hospit,lat,cm,second,go,work,around,fast,pain,woul | *"When I arrived at the hospital I was almost 5 cm and having contractions, but they just weren't strong or regular enough. Started pitocin at 7am and hard contractions started almost immediately. OW! Got epidural (pure heaven), water broke on its own and I was at 10cm and ready to push…she was born …No tearing/cuts, fast & easy, felt no pain. It was* *amazing. We left hospital after 24 hours and we're happy to be home with big brother!"* |
| **Recovery after birth** i,try,us,easy,rest,day,baby,expery,good,you,first,driv,red,thi,plan,tim,  numb,help,sleep,way,clear,famy,peopl,get,energy,hard,foc,today,real,  wor | "*I had my LO [Little One] last week. I’m feeling great now at the 2 week mark. The first week the pain was the worst. I’m glad I stayed 4 nights in the hospital cuz I was able to get more rest than if I came home.. are you taking the pain meds?"* |
| **Recovery after C-section** sect,c,recovery,surgery,walk,week,drink,he,first,wednesday,stretches,  diff,sex,birthday,tea,that,lat,lot,mis,weak,rrl,nettl,blend,upcom,ballgam,br,cholestas,icpc,icp,ursodio | *"I am 16 days pp [postpartum] from my 2nd c section. Dr told me the fastest way to recovery was to walk."* |
| **Arrival of new baby** congr,littl,born,girl,hom,on,joy,our,hun,bundl,glad,the,room,lil,sorry,  hug,tub,sunday,ppd,lbs,sound,contain,he,drug,best,deal,someon,anxy,  new,enoug | *“Congratulations on your new precious bundle of joy.”* |
| **Health of baby post-birth** i,baby,get,go,ear,week,know,hom,com,pregn,cal,help,told,could,the,  decid,right,lab,hospit,fin,day,if,sur,so,would,result,blood,end,don,du | *"So the morning me and my baby were to be discharged, she had a fever. The ped on call told us to wait because test and cultures had to be done to make sure she didn’t catch an infection. We were all worried until her temperature came down within hours and her blood work came back normal."* |
| **First few weeks** i,tim,week,lik,feel,baby,first,get,day,mak,last,help,know,pain,said,real,want,think,nee,husband,try,would,and,on,say,also,my,went,back,wai | "*In these early weeks things can all become a blur because they there are so many things close together. There's supposed to be a growth spurt at 2/3 weeks, Wonder Week at 5 weeks. It can be a rough time for parents thinking that things might go wrong or they might be doing something wrong. But in reality it's just an intense period of growth and development for your LO [little one]….. She's so adorable DH (dear husband) and I can't stop staring at her. Son is overwhelmed."* |
| **Breast- and bottle-feeding** bottl,fee,pump,milk,breast,formul,breastfee,nurs,nippl,supply,weight,  us,get,latch,the,suppl,much,eat,enough,gain,also,ount,increas,freez,  may,oz,giv,sometim,hungry,lac | *“There are some things you can also do to increase milk production, like fenugreek, drink Mothers Milk tea (it's at the grocer), eat oatmeal, brewers yeast, etc. Of course you can supplement with formula but if you don't need to (the baby is getting enough from you) then introducing formula may end up hurting your milk supply. If the baby isn't getting enough milk from you (weight gain would be a good indicator) then you SHOULD be supplementing. I would talk ALL of this through with a doctor and lactation consultant.”* |
| **Changes in family life and children's adjustmen**t daught,i,dear,lov,husband,dh,he,she,bath,dd,ad,it,feel,you,how,right,is,  tel,find,go,hop,old,am,though,excit,around,enjoy,hang,don,kee | *Since being home my DD [dear daughter] LOVES her new little sister and wants to help and do everything for her. She is so sweet towards her (holding her hands, saying I love your beautiful fingers, I love your beautiful hair LOL) BUT she is acting out too. She was the center of our universe before LO [little one] and now obviously has to share the spotlight. She is back-tracking with toilet training and purposely going in her pants. She is also running around and being super-hyper and won’t listen. If I warn her about a punishment that's coming she will make sure to do it again like she wants to be punished to get the extra attention. She also keeps taking LO binkies and crawling around acting like a baby. I am trying to handle it with patience and sitting her down and explaining that we still love her and making sure to give her one on one time but I can feel my patience thinning and with all these hormones from birth I know I'm going to end up snapping. Any advise is welcome! I want my sweet toddler back!* |
| **Management of two children** i,old,nap,baby,help,bed,toddl,she,month,nurs,giv,day,sist,my,tim,back,  night,he,girl,hour,week,newborn,put,lik,oldest,but,big,rock,feel,cri | *"I don't know what I'll do after next week when I'm on my own dealing with a newborn and a toddler that screams constantly, won't nap without 2 hours of cuddling, and then turns bedtime into a marathon nightmare scream-fest . . . Anyone else with a young toddler of the needy variety have advice to offer?"* |
| **Arranging life with two children** toy,smal,baby,beauty,i,old,tiny,mov,room,we,year,lik,stor,fig,new,put,  keep,toddl,tim,thing,plac,bit,spec,marbl,could,stil,worry,when,absolv,  aroun | *“We kept our son in our room in his own crib at first to make things easier at night. We then moved him when he was eight months into his own room. Now both boys have to share the room. I've been staying in there at night to make things easier and I probably will for at least the first six weeks.”* |
| **Concern over what is normal and what is not** would,us,if,thank,stinky,bp,giv,high,not,dry,urin,said,cardiolog,story,  told,lady,birth,think,ex,hear,real,pregn,d,bloodwork,horemom,everyth,  nev,somewh,test,no | *"Her umbilical cord is stinky… my daughter is 1 week old and it stinks bad! Is this normal? Please help! I know to keep it dry and not give baths, just sponge baths until it falls off. It's really stinky though!"* |
| **Baby's sleep** i,sleep,night,get,wak,month,us,old,week,baby,n,boy,stil,caus,happy,  think,lady,know,ev,cury,start,sometim,mon,would,alway,say,bad,three,real,watc | *"Mine rarely naps for more than 30 minutes at a time and he's also 5 months old. He sleeps well at night, but is a terrible napper. He's been like this since he was born.”* |
| **Baby digestion and feeding** eat,lik,he,oz,norm,proof,im,poop,someth,day,long,hrs,my,us,anyon,  baby,want,nippl,look,divorc,good,i,every,is,last,kid,put,thank,perfect,fi | *"I try to feed her every 3 hours but she’ll eat an oz or 2 and refuse anymore. She tends to eat about every 4 hours without problems. I am just worried it’s not good for her to eat so far apart."* |
| **Baby's general health** baby,giv,tim,she,sweet,try,want,we,od,oil,sur,lot,lov,good,chang,at,  acid,so,any,yeah,read,cut,much,i,wat,tomorrow,warn,room,indigest,hel | *“My baby is about 4 months old and has acid reflex whenever she has milk….. sometimes she spits alot ... is there anyone in the same situation and have any suggestions?”* |
| **Returning to work** i,on,littl,lo,my,she,day,try,work,tak,us,good,old,also,it,back,luck,son,  mak,around,going,help,put,tri,baby,sur,week,tim,lik,kee | "*Anyone else going back to work this week? I forgot how hard this transition is. I cried a lot this week! I wish there was more time, but I'm grateful for every minute of my maternity leave with my little guy. Also...I miss my maternity pants! Normal pants are so uncomfortable and not stretchy! Lol"* |

*Note.* The label of the LG topic was generated through consensus and is displayed along with tokenized keywords from the LDA model. LG’s are listed in temporal sequence starting with posts in the month of labor and delivery and ending with posts in the later postpartum period. To protect the privacy of individual posters, each LG includes a lightly anonymized quote from a post as an example.
